# Contract doctors’ strike in Malaysia: A content analysis of the perception of medical fraternity and stakeholders on Facebook

**DOI:** 10.1371/journal.pone.0292213

**Published:** 2023-09-28

**Authors:** Norehan Jinah, Kun Yun Lee, Nor Haniza Zakaria, Nursyahda Zakaria, Munirah Ismail, Shazwani Mohmad

**Affiliations:** Centre of Leadership and Professional Development, Institute for Health Management, National Institutes of Health, Ministry of Health Malaysia, Selangor, Malaysia; Sunway University, MALAYSIA

## Abstract

Contract appointment policy for newly graduated medical officers was implemented by the Ministry of Health Malaysia in 2016 to overcome the lack of permanent posts. Contract officers faced disadvantages in terms of salary, leave provision, and career prospects. A nationwide strike, *Hartal Doktor Kontrak* (HDK) was organised on 26^th^ July 2021. Besides generating widespread public attention, HDK was also closely scrutinised by the medical fraternity and stakeholders. This content analysis aimed to explore how the medical fraternity and stakeholders viewed the strike as their perception would offer vital insights into the fundamental causes and viable solutions to the contract appointment policy. A qualitative content analysis of Facebook (FB) posts on the HDK strike was conducted from 1st June 2021 until 28th February 2022. A total of 182 FB posts were retrieved from stakeholders, medical fraternity groups, and medical key opinion personnel. Inductive coding was used in the thematic analysis to identify pertinent themes. Three main themes emerged: triggering factors, reactions to the strike, and outcomes of the strike. Factors that led to the strike included unequal treatment faced by contract officers, frustration with the government’s lack of long-term solutions, and aggravation by the COVID-19 pandemic. In terms of reactions, there was a mixture of supportive and opposing voices. No substantial negative impact on the healthcare service resulted from the strike. Instead, it generated widespread attention that propelled the government into implementing solutions to prevent adverse short and long-term consequences. Various suggestions were proposed, including the reform of human resource planning and undergraduate medical education. The results highlight the importance of proactive systemic measures by the government to prevent further strikes that may jeopardise healthcare provision. In summary, social media was found to influence the progress and outcome of HDK, thus demonstrating the impact of media influence on similar issues.

## Introduction

Many countries frequently use strikes as a legitimate deadlock-breaking mechanism when labour negotiations between affected parties fail during collective action. Each strike is motivated by a distinct combination of local conditions, employee concerns, and most importantly, the relationship between employers and trade unions. The international health community is becoming increasingly concerned about healthcare worker (HCW) strikes due to the effect on healthcare service delivery [1]. HCW strikes are frequently the results of failed employer-employee negotiations on fair wages and working conditions, policy issues, a lack of infrastructure, and concerns about personal safety in the workplace, particularly in developed countries such as the United Kingdom (UK), the United States (US), Israel, and Norway [2]. In 2012, the British Medical Association (BMA), which represents two-thirds of UK medical practitioners, organised a non-urgent care boycott in response to government pension reform in which doctors would need to work longer hours but receive fewer retirement benefits [3]. Aside from that, Irish doctors staged a protest over disagreements over long working hours, which violated EU employment law and could endanger patients [4]. During the COVID-19 pandemic, there were also HCW strikes in Hong Kong, and the US in response to various issues with outbreak management [5]. Strikes like these by healthcare workers can sometimes lead to detrimental impact on the service delivery and patient care.

A strike is almost unheard of in Malaysia, especially in the healthcare setting. The last two strikes by doctors in the country, both precipitated by wage-related issues, dated more than 40 years back in 1977 and 1982 [6]. Since the days of independence, medical officers have always been given permanent appointments. However, beginning in 2016, the Malaysian government enforced the contract appointment policy for house officers due to a lack of permanent medical officer positions available. This followed the directive of the Public Service Department (JPA) to halt the formation of new posts in all governmental agencies in accordance with the Malaysian Government Policy on human resource optimisation [7]. The five-year contract allows medical graduates to fulfill their mandatory service requirements (three years as house officers and two years as medical officers). However, between December 2016 and May 2021, only 3.4% out of the 23,077 contract medical officers were given permanent appointments in the Ministry of Health (MOH) following the completion of their five-year tenure [8].

On 23^rd^ June 2021, a group known as the *Hartal Doktor Kontrak* (HDK) was formed to fight for the rights of contract doctors. It consisted mainly of junior contract doctors who shared the same plight. The HDK movement used social media to rally support for the plight of contract doctors across the country, as well as to voice their demands in terms of policy change to the government. and they eventually called for Malaysian doctors to go on a strike, i.e. “Hartal” (a mass protest which brought all commercial activity to a standstill for a limited period). The protest, which was expected to culminate in a massive labour strike, took place on 26^th^ July 2021. The massive dissemination of the event across both traditional and social media drew widespread attention from the medical fraternity, the general public, and stakeholders. It was no longer confined to an internal MOH issue but had become a national public concern. Various healthcare groups and associations, as well as individual medical personnel have expressed their viewpoints on many platforms, most notably on social media. As a result, our study aimed to explore the perception of the medical fraternity and stakeholders towards the HDK strike in order to better understand the underlying issues of contract appointment policy to provide practical recommendations for solving the issue.

## Methods

This was a qualitative content analysis of Facebook (FB) posts focused on the issue of the HDK strike from 1^st^ June 2021 until 28^th^ February 2022. In this content analysis, we purposively sampled FB pages of three main groups, namely government stakeholders (SH), healthcare interest groups (HIG) such as medical association/ network/ platforms, and lastly prominent medical key opinion personnel (MP). All these FB pages were set as ‘PUBLIC’ and thus the posts were visible to all FB users at the time of study. For the group of MP, senior healthcare personnel, i.e. specialists or experts in their respective fields who frequently write and share their opinions related to health systems on their public FB pages with substantial amount of FB followers were selected.

The content of the relevant FB posts formed the data for this study. The keywords "contract doctor", "hartal", “HDK” and "strike" were used in the search. As this content analysis focused on written texts, only primary FB posts in the forms of sentences and paragraphs that were written by the owners of the FB pages were included. Other forms of posts such as photos, reels, video clips, links, webpage link, polls, infographic posters that required different analysis methods were not included for the purpose of this study. FB posts shared by the owners from other sources were also excluded.

In total, 182 posts were extracted from the official FB pages of three selected SH: i. MOH Malaysia, ii. Minister of Health, and iii. Director-General of Health; four selected HIG: i. Section Concerning House Officers, Medical Officers, and Specialists of Malaysian Medical Association (SCHOMOS MMA), ii. CodeBlue, iii. Health Empowerment and Reform Team (HEART), iv. Malaysian Primary Care Network (MPCN); as well as seven prominent MP in the country with FB followers ranging from 10,000 to 638,000.

We performed a thematic analysis of all the transcripts from the related FB posts based on the six-step framework described by Braun and Clarke [9]. All relevant posts that covered all aspects of the study aim were identified and extracted. After completing data collection, one researcher performed data cross-checks and data cleaning. Duplications were identified and eliminated. After all the researchers familiarised themselves with the data and agreed on the preliminary coding approach, the inductive process of content analysis was performed. The transcript data were inductively coded until saturation was achieved. Open coding was conducted by two researchers at the initial stage to form a codebook. The final codebook consisted of codes agreed upon by all the researchers. Meetings were held regularly to discuss the abstraction process in formulating categories and themes that contributed to answering the research questions. Discrepancies were resolved through a series of discussions among all the investigators. NVivo 12 was used for data management and analysis.

We acknowledge that the experiences and opinions of the researchers may have influenced data analysis and caused unintentional bias. However, all researchers had a strong background in medical research and had also received training in qualitative research, including content analysis courses. They also had common experience conducting prior research regarding contract house officers, so they shared a common perspective and understanding of the issue of interest. Furthermore, any bias was explicitly examined throughout the generation of codes and themes.

This study was registered under the National Medical Research Register (NMRR) of Malaysia (NMRR ID-22-00850-MTS). The Medical Research and Ethics Committee (MREC) of the Ministry of Health Malaysia exempted ethical approval for this study as it does not involve human subjects. Based on similar studies in the literature [10], the justification for accessing and analysing data from FB was that users’ data exists in the online public domain and can thus be accessed by anyone. In other words, the data in this study were all retrieved from a public space as shared by the respective owners of public FB pages who understood that they should not have a “reasonable expectation of privacy” with regard to their posts.

## Results

The three main themes and sub-themes derived from this qualitative thematic analysis are presented in Table 1, including (1) triggering factors of the strike, (2) reactions toward the strike, and (3) outcomes of the strike.

**Table 1:** Summary of identified themes and sub-themes.

Theme 1: Triggering Factors of Strike	Theme 2: Reactions toward the Strike	Theme 3: Outcomes of the Strike
1.1 Inequitable treatment of officers under the Contracts Appointment Policy	2.1. Support toward the organisers before the strike	3.1. Direct effects of the strike on the healthcare service
1.2. Frustration with the lack of long-term solution from the government	2.2. Opposing voices toward the strike	3.2. Urgency of taking effective actions
1.3. Exacerbation by the COVID-19 pandemic	2.3. Suggested alternatives as substitutes to the strike	3.3. Suggested solutions and ways of achieving amicable solutions

### Theme 1: Triggering factors of strike

Many factors can precipitate a work strike. In the healthcare setting, it is often provoked by poor remuneration, high workloads, and long working hours. Our analysis identified several unique triggering factors for this HDK strike in Malaysia.

#### 1.1 Inequitable treatment of officers under contract appointment policy

Many parties lamented the discrimination faced by contract doctors under the contract appointment policy. The differential treatment is especially stark when compared to their predecessors who received permanent appointments in the government sector. As expressed below, many highlighted the plights of these officers and their rights to be given the same treatment as their permanent counterparts.

***What are these rights? The right to equal salary and benefits, the right to postgraduate training, the right to a secure and certain future, and the right to equal treatment by the administration and colleagues.** (HDK218, HIG1)*

*…**its** (contract doctor hiring system’s) **execution has been dismal and fraught with issues**. **Our juniors on contract continue to be denied their basic right to equal pay, equal welfare benefits, and equal career opportunities**. (HDK197, HIG2)*

Previously, after completing housemanship, house officers would be automatically integrated into the civil service as permanent medical officers to a higher salary position (grade UD44). However, under the contract appointment policy, they would remain at grade UD41 (lowest starting salary position for medical doctors), thus giving rise to a pay discrepancy of at least RM8,000 annually for the same work level as their UD44 counterparts. Additionally, they were ineligible for the government’s scholarship for specialisation, as well as entitlement to unrecorded leaves and government loans.

*The current contract system places qualified MOs on **a lower pay grade relative to doctors in permanent posts**. They also **have fewer benefits, such as limited leave allowance, including for study, and exclusion from applying for postgraduate training scholarships**. (HDK213, HIG2)*

#### 1.2 Frustration with the lack of long-term solution from the government

The frustration of contract doctors increased following short-term and reactive solutions offered by the government, especially after a series of discussions with stakeholders hit a dead end. Upon completing the five-year term, only a limited number of contract doctors were offered permanent positions in the MOH based on the availability of posts.

*Khairy (Health Minister) said the government’s contract doctor system, which was first introduced in 2016, has **only managed to absorb a total of 1,118 medical officers** to permanent roles, up until 2021. (HDK222, HIG1)*

Even though the government took measures in 2020 to raise the pay scale for contract officers from UD41 to UD43 (instead of UD44), the action did not entirely resolve the salary issues as they would reach a ceiling salary with no time-based increment like their permanent counterparts.

*Amongst what we (SCHOMOS) have taken note include **the grade offered is UD43 after 5 years of service which is not equal to what was offered previously**. (HDK200, HIG2)*

*As a contract doctor, her **monthly salary of about RM5,000 will stagnate** and will not be enough to support her family… (HDK214, HIG1)*

According to a few HIGs, this contract policy was interpreted to be detrimental as it offered almost no job security and minimal opportunities for contract doctors to advance in their careers.

***These contract doctors are constantly on tenterhooks, due to the lack of job security and opportunities for career progression**… (HDK208, HIG1)*

*….**a one-year extension of the contract is not a definitive solution to the problem**. The **frustration of these junior doctors is growing fast** into a rage of fury as the clock ticks with **no job security or a career plan.** (HDK143, HIG2)*

Moreover, many also questioned the lack of fulfilment of the promises to absorb contract officers into permanent posts after the end of their contracts. As these issues remain neglected, the call for a strike should be viewed as a cry for help to reignite the discussions about it.

*Contract doctors want life stability, transparency, and career advancement….**it used to be said that "permanent post" opportunities will follow merit. But where is it all? Promises are just promises.** (HDK 230, MP3)*

*Our pleas and appeals have been falling on deaf ears for five years. **The time of diplomacy is over. We must act now.** (HDK21, HIG1)*

#### 1.3 Exacerbation by the COVID-19 pandemic

With the emergence of the COVID-19 outbreak, the demand and burden on healthcare sectors increased tremendously as the country struggled to control the infection. The contract doctors were amongst those deployed to battle the pandemic at the frontline. As the waves of COVID-19 continued to escalate, the frustration among the contract doctors grew as the remuneration they received was thought to be not comparable to their efforts.

***Contract doctors, the majority of whom are involved as front liners in the fight against COVID-19**, have been facing uncertainties about their future. (HDK151, HIG2)*

Similar to other medical personnel, contract doctors bore a tremendous burden. This was significant in light of a Malaysian study that found that more than half of them felt burned out while on duty during the pandemic [11]. Furthermore, drastic changes in workload, staffing levels, relationships with colleagues and patients, lack of family support, and uncertainty surrounding the workplace all led to feelings of discouragement and stress, as mentioned below by the HIGs.

***Many young doctors are experiencing adverse mental health problems, and this contract vs permanent employment issue, coupled with high work demands will contribute to the development of depression, anxiety, and stress**. (HDK208, HIG1)*

*Contributing factors (suffering from burnout) could be **workload expectations, fear of infection, a toxic work environment, sleep deprivation**… biological hazards. (HDK210, HIG1)*

*MMA (Malaysian Medical Association) urged the Prime Minister to make a decision **as soon as possible** as these frontline doctors during this pandemic have been **totally demoralised and demotivated**. (HDK173, HIG2)*

### Theme 2: The reactions toward the strike

Following the announcement of the strike by HDK, there were various responses from the stakeholders, HIGs, and MPs. The responses were a mix of supportive and opposing voices, with some of them offering alternative suggestions.

#### 2.1. Support toward the organisers before the strike

Some HIGs and MPs related the HDK strike to those that have occurred in the past and shared anecdotes. They quoted various reasons for their support of the strike action.

*A strike is not uncommon. **In other countries, a union would organise the strike**. Was patient care jeopardised during the 2015 NHS junior doctors strike in the UK? No, because it was an organised strike that only took one day. (HDK210, HIG1)*

*When I was a consultant in the UK, **the junior doctors in England were planning a strike all across England because of an unfair new doctor’s contract proposed by UK Secretary for Health**. Many consultants supported our junior doctors. We fought hard for them and we had their backs. (HDK241, MP4)*

*……**but I believe their strategy is the way to do it, which is a strike**. You can’t win wars without a fight. (HDK214, HIG1)*

*.….the number of doctors in the country is still not enough, **the defence movement of contract doctors is justified and the hartal movement is justified**. (HDK227, HIG4)*

#### 2.2. Opposing voices toward the strike

However, other HIGs were concerned about the call for a strike, especially since it came at a critical time of the pandemic with an increasing patient load and depleting hospital capacity across the country. Many feared that this strike would cripple the country’s public healthcare system.

*The MMA wishes to clearly state that it is working with the authorities to resolve all issues concerning contract healthcare workers. **We DO NOT condone any strike by any healthcare professionals, especially during this time of a pandemic**. (HDK207, HIG1)*

*Why don’t I support a work strike? **A work strike goes against the very Oath we have taken to care for our patients**. It does not and will not help our cause of working towards a better career pathway for our junior doctors. (HDK156, HIG2)*

*This strike might lead to complications, worsening the situation in the fight against COVID-19, specifically when **there is the possibility of compromises in patient care**. (HDK209, HIG1)*

Conducting a strike action would appear to directly contravene the code of ethics. Therefore, the MOH also released a statement, asserting that those participating in the strike may face disciplinary actions for violating the Medical Act 1971.

*The Health Minister **stressed that a strike is against the law and those involved risk disciplinary action** and being struck off the register by Malaysian Medical Council (MMC). (HDK163, HIG2)*

#### 2.3. Suggested alternatives as substitutes to the strike

As an alternative to the strike, SCHOMOS organised Code Black, a protest initiative in which contract doctors were encouraged to wear black to work between the 1^st^ and 12^th^ of July 2021, as a show of solidarity in the fight for contract doctors’ rights.

***Code Black (emergency code)–a hospital emergency code denoting a threat to personnel–in this case, our contract healthcare workers**. This is a way to show support for them. (HDK207, HIG1)*

***Our calls for a Code Black and culminating in a Black Monday is a show of unit**y, to inform the politicians to take action as soon as this matter has progressed to a stage, we will need the support of the Cabinet for its approval. (HDK237, MP1)*

### Theme 3: The outcomes of the strike

The direct impact of the strike on the healthcare service and patient care was a great concern for many before the event. However, the contract doctors viewed the strike as a conduit for their demands to be heard and addressed by the stakeholders. The thematic analysis shows that the strike did not produce any substantial negative impact on the healthcare service. Instead, the strike gained the necessary attention to propel the government into seriously looking for permanent solutions for the contract appointment policy.

#### 3.1 Direct effects on the healthcare service

The HDK strike was well-coordinated and only lasted a few hours. The contract doctors walked out after handing over their ward duties to their colleagues, carrying placards, and undertook a peaceful protest. As opposed to the concerns of some that the strike might compromise patient care, no direct interruption in the healthcare service was observed.

*There were **no reports of any casualty, neglect, or negligence towards any patient during their short walk out of the hospital** to highlight their grievances. **They made sure there was cover during the walkout**. (HDK125, HIG3)*

***The protest by contract doctors today was brief, and without any reported disruptions to the health of patients**. (HDK179, HIG2)*

#### 3.2. Urgency of taking effective actions

Following the strike, critical and active measures must be taken immediately to address the underlying human resources issues related to contract appointments, which was described as a national emergency by one of the posts. As COVID-19 continues to spread, it is even more of a top priority to implement viable solutions for contract doctors or risk losing our doctors to other countries.

***It cannot be more obvious that our manpower issues must be addressed urgently. Facilities and equipment can be acquired quickly within days, but human capital requires decades of meticulous nurture**. Even as we are fighting the pandemic, scores of doctors are forced to **leave the country to seek better job** prospects. (HDK173, HIG2)*

*They are bright gems and will be the future of Malaysia’s medical care. This pandemic has shown how much we need them. **Without them who form 60% of our workforce in many departments, our healthcare will collapse.** (HDK247, MP2)*

The main demands of the HDK movement included a higher number of permanent posts and clearer career progression. The failure to meet their demands may lead to consequences such as specialist shortages in the near future. This was highlighted by a few different sources. Worse still, it may leave them with poor job security, provoking them to leave the service or the country, thus leading to brain drain in the long term.

***Obstruction of these contract medical officers’ specialisation routes** will lead to a shortage of specialists in the future. (HDK208, HIG1)*

*Wait a few more years, and we will **suffer from the double blows of even higher costs and greater brain drain**. Bear in mind that our Malaysian **population-to-specialist ratio is 10000:3.88**. This is embarrassingly low when compared to the ratio of 10000:14.33 in OECD countries. (HDK173, HIG2)*

#### 3.3. Suggested solutions and ways of achieving amicable solutions

The HDK strike generated more intense attention from the public toward the plight of contract doctors. Various solutions to resolve the controversies surrounding the contract appointment policy were put forth by various individuals and organisations. On a higher level, reform of human resource planning in the public healthcare system was proposed by certain stakeholders.

*High-income countries continuously **reform their healthcare system.** …. Among the reforms that they have implemented are the **strengthening and increase of staffing levels**… (HDK208, HIG1)*

*The government **must expedite the restructuring process of the civil service with a rigorous rationalisation of positions and job scope. Rapid digitisation has rendered some jobs obsolete**. Warrant of Appointment awarded for these positions can be apportioned to other ministries such as the MOH, in order to create more permanent positions. This can also reduce any financial implications for the government if more permanent positions are created. (HDK208, HIG1)*

*We justified the increase in positions to be tied to the growth of the population and the increasing health needs that would follow. We have discussed this and **Economic Planning Unit has commissioned the study to see the actual workforce needs for healthcare**. (HDK156, HIG2)*

Apart from the calls for the revamp of the healthcare system, some parties also voiced the need to focus on the high number of medical graduates produced by local and foreign medical schools.

*More stringent regulations have to be implemented to ensure this large influx of medical graduates is kept in check. **The government should identify medical schools that are admitting students who do not meet minimum requirements and de-recognise these programmes**. (HDK208, HIG1)*

***Tripartite committee (MOHE, MMC, JPA) for the evaluations** on medical program & schools quality standards & numbers years. (HDK289, SH3)*

On the other hand, to solve the predicament faced by contract doctors who are already working in the MOH, there is generally a strong push by all the different parties to convert them into permanent appointments. However, many opined that detailed eligibility criteria and a transparent process should be put in place to adequately reward those who meet a benchmark performance level.

***Promotion must be based on performance, not time based and transparent**… Absorb them into permanent posts in accordance with needs and MUST be transparent. Go through the process: real interviews, CVs, and marking schemes… Not all should be absorbed. (HDK249, MP3)*

*The better solution is 1) **Contract extension** for all the affected doctors, 2) **Absorb them into a permanent position**. The option is—permanent with Employees’ Provident Fund, 3) **Same entitlement and opportunities as the permanent officers**. (HDK263, MP6)*

Nevertheless, this process cannot be instantly executed as it involves layers of administrative procedures and approval. Thus, it is also of popular opinion among many HIGs that alternative solutions should be considered in the meantime, such as extending the existing contracts. Furthermore, it is proposed that contract officers be given the same access to specialisation and paid study leave as their permanent counterparts.

*There should be **an automatic ten-year contract awarded to graduates to complete their compulsory training and consequently begin postgraduate and specialisation pathways**. (HDK213, HIG1)*

*….Cabinet to consider all our demands and resolve this issue once and for all: (1) **provide clear postgraduate pathways to specialisation for all doctors**; (2) publish a detailed and transparent ranking system for appointment to permanent positions; (3) provide equal and fair treatment for contract and permanent staff; and (4) assure job security for all contract doctors. (HDK167 & HDK173, HIG2)*

With the threat of a second strike looming, suggestions were put forth for a collaborative meeting between all the parties of interest to formulate viable long-term solutions for the contract appointment issues surrounding healthcare professionals in the shortest time frame.

*I (Health Minister), **together with the Secretariat of the ‘Hartal Doktor Kontrak’ Movement of Contract Doctors, listened to the issues and problems faced by them**. (HDK 291, SH2)*

*We **had a town hall session** with Health Minister today to discuss the various issues about doctors in the country. It was indeed the first time that we engaged with MOH, MOF, and JPA in a town hall session. We were also **joined by colleagues from Gerakan Hartal, MMI, and IMAM** who were there to express grievances of doctors in Malaysia. (HDK204, HIG2)*

To reciprocate, the government has also extended an open arm to include other stakeholders in the planning process for better representation and for voices from the ground to be heard.

*Prime Minister has committed **to inviting MMA to the MOH Policy Making Committee as a key stakeholder** to allow for better representation. (HDK176, HIG2)*

## Discussion

A strike is identified as a collective action that generally involves a temporary interruption of work to voice certain demands for the economic benefit of the strikers [12]. Sylvester and Chima [1] mentioned that strikes are strategies used by an employee or group of employees as leverage for the employer to meet their demands. Among HCWs, the determinants of strikes can be diverse. In the past, HCW strikes mostly stemmed from dissatisfaction over salaries, working hours, and other administrative factors [13]. While it is necessary to understand the underlying factors that provoke the call for strike by the organiser (i.e. employee/ staff), it is also important to explore the perceptions towards the strike among the medical fraternity and stakeholders (i.e. government/ employer/ influential personnel) with an active and participatory role in making a decision or providing a solution.

This study highlighted the three themes that emerged from the wide range of perceptions towards the HDK strike among SH, HIG, and influential MP, namely the perceived triggering factors, responses toward the strike, and lastly, the perceived impact of the strike. The inequitable treatment between contract officers and their permanent counterparts in the MOH sparked off the dissatisfaction that gradually brewed into prolonged frustration, especially when the solutions offered were viewed as reactive and short-term in nature. The situation took a turn for the worse when these officers bore the tremendous burden as the front liners in the COVID-19 pandemic and more than half of them experienced burnout [11]. Nevertheless, they felt unappreciated and demotivated as no viable solution was offered by the government about their contract employment status. Their job security and career pathway were also unclear. All these interrelated factors prompted some of the contract doctors to call for the HDK strike. In a way, the strike became the last resort here as a deadlock-breaking mechanism as they felt that the negotiations had reached an impasse during collective bargaining [2, 14]. Similar strikes happened in the UK before to fight for better pay for junior doctors as well as in Hong Kong, and the US during the COVID-19 pandemic due to mismanagement that adversely affected HCWs [5].

Contrary to other professions, HCW strikes often attract extra public attention due to the underlying ethical dilemma [15]. Boycotting the work of delivering care and potentially compromising the safety of needy patients are viewed as violating the Hippocratic Oath, the primary moral consideration in medical practice [2]. Based on the FB posts of stakeholders, the HDK strike was condemned because it was against the code of ethics with a potentially adverse impact on health services. Likewise, strikes are prohibited by almost all medical councils in the world. For instance, the Delhi Medical Council in India stated that "Doctors should never resort to strike action" [16]. On a similar note, doctors in the UK are required by the General Medical Council (GMC) UK to make sure that the industrial action or strike does not endanger patients. The American College of Physicians (ACP) also opposes any sort of work stoppage, even when it is aimed at prompting healthcare system reforms to address infrastructure issues, poor policy, or poor governance [17]. In comparison, even though the American Medical Association (AMA) allows some methods of collective bargaining, the code of ethics explicitly forbids doctors from using strikes as a means of negotiation [18]. According to a retrospective analysis of recent doctor strikes in the UK [3, 19], New Zealand [20], and Kenya [21], the main effects of doctor or HCW strikes are disruptions in the delivery of healthcare services to the general public, such as decreased elective admissions, increased outpatient cancellations, fewer admissions, and fewer accident and emergency attendances but there was no significant rise in patient mortality during HCW strikes [17, 19, 21, 22]. Nevertheless, these studies were conducted in the pre-COVID era.

In the case of the HDK strike, the contract doctors saw the strike as the last resort for their plights to be given serious attention by the authority in power. In general, our findings revealed that almost all HIGs and prominent MPs supported the strike in solidarity with the contract doctors. In other countries, HCWs’ support toward strike action was divisive and frequently fragmented [13]. For example, it was widely supported in Israel [23] and Croatia [24] while frowned upon in Nigeria [25]. However, it was a critical period to call for a strike among front liners, especially when the country was facing an unprecedented crisis due to the COVID-19 pandemic and the healthcare system had been stretched to the maximum. Any disruption of service delivery that compromises patient care could risk the ire of the public and backfire on them, possibly diminishing the public support for their cause. Considering this, the strike was planned as a brief walk-out after a proper handover of their tasks at work. There was no long period of work boycott or protest march on the streets. As commented by the medical fraternity, there were no disruptions to the health service. Judging from the subsequent responses, the peaceful protest worked out to the benefit of all who were involved.

In a way, the strike also acted as a catalyst for expedited actions. The underlying issues were not solved by reactive measures like short-term contract extensions and wage readjustments. Even worse, it might jeopardise the health system’s robustness [26]. Despite different views, all parties mutually agreed to cooperate in brainstorming and implementing the most appropriate mechanisms to solve the crisis faced by these contract doctors. Following the strike, a special task force led by the MOH and Malaysian Medical Association (MMA) was set up in September 2021 to formulate viable long-term solutions for all the issues of contract appointment policy. Some suggestions that were put forth included the possibility of amending the Pensions Act 1980 and utilising the Employees Provident Fund (EPF) scheme to offer permanent jobs for contract officers. This was similar to what happened in Kenya when the Musyimi Task Force, composed of six government officials and six union members, was formed in 2011–2012 to address concerns about the health sector, particularly poor working conditions and low wages [27]. In addition, the newly appointed Minister of Health held a town hall session with representatives of contract doctors and several medical associations in the presence of other stakeholders such as JPA and the Ministry of Finance to obtain multi-faceted input on this issue. Russo et al. [28] found that when other ministries (such as the Finance or Public Administration Ministries) or higher levels of decision-makers (such as the Prime Minister or President) were involved in the process, resolutions were more frequently reached as compared to actions taken by a single ministerial department. In this case, issues pointed out were acknowledged by the stakeholders and some of the recommendations were implemented as a system-wide effort to address the plights of these doctors. To begin with, their work contract was extended by up to four years to allow for specialisation. Additional permanent posts were also created. Benefits such as paid study leave, specialisation scholarships, flight warrants, and other leaves were extended to contract doctors as well. These were widely shared on the FB of SH, HIG, and MP alike and garnered positive reception from all the involved parties, giving temporary relief to the contract doctors.

Nevertheless, from a wider perspective, the HDK movement highlighted the importance of long-term solutions to be put in place as the contractual employment policy may have larger repercussions not just for the future of medical practitioners, but also for the healthcare system and population health. A similar sentiment was echoed in another study from Kenya [29]. Many of the HIG and MP warned that the existing brain drain among the medical profession will worsen with a massive exodus of contract doctors if they sense a bleak future with a lack of job security and career progression. As illustrated by the WHO building blocks of a healthcare system [30], the health workforce, together with the other building blocks represents the vital inputs to ensure equitable access, coverage, quality, and safe healthcare for the people. Thus, it is not surprising that our findings showed that HIG and MP were mostly in support of the HDK strike. A continuous supply of trained healthcare workforce in the system relies on the provision of a better working environment and guaranteed job security. In Malaysia, the recruitment of public servants is centralised under JPA. MOH, as the major public healthcare provider and one of the biggest ministries in the Malaysian government, was debilitated by the freeze of the creation of new posts in the public service in 2015. The serious understaffing issues in MOH hospitals were also reported by the Department of Audit in 2018. However, without additional funding from the Ministry of Finance (MOF) and JPA, MOH is unable to unilaterally recruit more HCWs. As a result, this has led to calls from certain groups to establish a separate pathway and agency responsible for the recruitment of HCWs under the direct jurisdiction of MOH. Nevertheless, this might not materialise soon considering the prevailing economic and political circumstances. In other words, health human resources play a vital role in the delivery and output of healthcare services. Thus, apart from taking immediate actions to tackle any ongoing human resource issues, long-term meticulous planning is crucial to ensure the sustainability and seamlessness of patient care. The HDK strike during the pandemic is a clear imperative to improve healthcare human resource management and to develop proactive policies and risk-management plans that can strengthen the systems’ ability to withstand crises while continuing to function [26].

There are some limitations to our study. Firstly, we were unable to portray all frame elements completely because our samples were limited to selected FB groups and pages. The FB pages included in this study were purposively sampled to identify the representative voices of the largest and most influential healthcare groups and individuals in Malaysia. In addition, we did not explore the perceptions of affected contract doctors as it was not within the scope of our study objective. Furthermore, because of the nature of the study design, the level of subjective interpretation can affect the reliability and validity of the results, resulting in potential cognitive bias.

## Conclusion

A strike by HCWs in Malaysia poses various unprecedented challenges, particularly during the pandemic. It emphasises the need for proactive measures to be put in place as reactive responses would only provide short-term reprieve. Although the MOH has taken some steps regarding contract doctors, more comprehensive long-term actions by all the stakeholders involved are necessary to address the root causes of the contract employment issues. Left unaddressed, recurring HCW strikes may cripple healthcare services and compromise the health and well-being of the people. The government’s roles and responsibilities in a devolved health system should be clearly defined to prevent similar strike incidents in the future. Mechanisms for dispute resolution should be established by anticipating and pre-empting changes in the health system that may result in conflict between parties. Both sides must remain rational and fair, whereby the demands made by HCWs should be reasonable and the government must respond in a suitable manner, especially in a complex setting like the healthcare system. Furthermore, social media has been shown to influence every step of the strike action process in this case, thus the role and utilisation of the media in covering similar issues should not be overlooked.
